# No Survival Improvement for Patients with Angioimmunoblastic T-Cell Lymphoma over the Past Two Decades: A Population-Based Study of 1207 Cases

**DOI:** 10.1371/journal.pone.0092585

**Published:** 2014-03-20

**Authors:** Bei Xu, Peng Liu

**Affiliations:** 1 Department of Medical Oncology, the First Affiliated Hospital of Nanjing Medical University, Nanjing, PR China; 2 Department of Hematology, the First Affiliated Hospital of Nanjing Medical University, Nanjing, PR China; National Cancer Center, Japan

## Abstract

Angioimmunoblastic T-cell lymphoma (AITL) is a rare lymphoid malignancy with dismal prognosis. We conducted a large population-based study using the Surveillance, Epidemiology, and End Results (SEER) database (1973–2010) to determine the temporal survival trends and prognostic factors of AITL patients. A total of 1207 patients with AITL were included in this study, with a median age at diagnosis of 69 years. At presentation, most patients (79.5%) had an advanced-stage disease. Overall survival (OS) probabilities at 2, 5 and 10 years were 46.8%, 32.9%, and 21.9% respectively. Two-year, 5-year, and 10-year disease-specific survival (DSS) rates were 56.1%, 44.0%, and 35.9% respectively.On multivariate analysis, age older than 70 years, advanced-stage disease and male sex were identified adverse predictors for OS and DSS. We failed to find any survival differences among subgroups diagnosed in the 5 periods studied (1992 to 1998, 1999 to 2001, 2002 to 2004, 2005 to 2007, and 2008 to 2010). The current study represents the largest specific series of patients with AITL and the first investigation on temporal changes in survival of AITL patients. There has been no survival improvement for AITL patients over the past two decades. Further investigations are warranted to develop more effective treatment for AITL.

## Introduction

Angioimmunoblastic T-cell lymphoma (AITL) is a rare lymphoid malignancy, accounting for approximately 2% of all non-Hodgkin lymphomas [1]. AITL represents the second most common form (18.5%) of peripheral T-cell lymphoma (PTCL) worldwide [2], [3]. It displays an aggressive course and is clinically characterized by sudden onset of constitutional symptoms, generalized lymphadenopathy, hepatosplenomegaly, anemia and polyclonal hypergammaglobulinemia. Also, this disease is frequently associated with autoimmune phenomena, such as hemolytic anemia and thrombocytopenia [1].

The standard approach for treating patients with AITL has not yet been clearly established. Treatment options consist of steroids, immunomodulators, single-agent cytotoxic drugs as well as combination chemotherapy [1], [2]. Of these, Anthracycline-based chemotherapy has been recommended as first line therapy. Additional treatment modalities have been employed to obtain better outcomes, including novel agents (Alemtuzumab [4], Bortezomib [5], rituximab [6], histone deacetylase inhibitors [7], antifolic drugs [8], etc.), more intensive chemotherapeutic regimens, consolidation with autologous stem-cell transplantation (ASCT) [9], [10], and even allogeneic stem-cell transplantation (alloSCT) [11]–[13]. However, there is little evidence from randomized controlled trials that these therapies have improved survival of patients with AITL. Several retrospective studies were conducted, with reported 5-year overall survival (OS) rates ranging from 25% to 41% (Table 1). Because of the rarity of AITL, all these series based their conclusions on limited numbers of patients (range, 45 to 243).

**Table 1:** Large series of patients with AITL reported in the literature.

Study	No. of Patients	Median age, years	Stages III to IV, %	Overall survival, %
Siegert 1995 [14]	62	64	90	36 at 4 years
Park 2007 [15]	65	60	95	25 at 5 years
Mourad 2008 [16]	157	62	81	51 at 2 years; 33 at 5 years; 29 at 7 years
Kyriakou* 2008 [10]	146	53#	73∞	67 at 2 years; 59 at 4 years
Kyriakou§ 2009 [13]	45	48	69	66 at 1 years; 64 at 3 years
Tokunaga 2012 [17]	207	67	90	54 at 3 years; 41 at 5 years; 35 at 7 years
Federico 2013 [18]	243	65	89	33 at 5 years

**Abbreviations**: AITL, angioimmunoblastic T-cell lymphoma.

*Patients were treated with high-dose therapy followed by autologous stem-cell transplantation.

# Age at stem-cell transplantation.

∞ Stages IV, %.

§ Patients were treated with allogeneic stem-cell transplantation.

Administrative data sources, which provide access to large numbers of patients, are particularly well suited for the study of rare diseases such as AITL. Using data from the Surveillance, Epidemiology, and End Results (SEER) program, we evaluated the temporal survival trends in a large population-based cohort of AITL patients in the United States and determined the prognostic factors of this disease.

## Subjects and Methods

### Study Population

**Figure 1:**
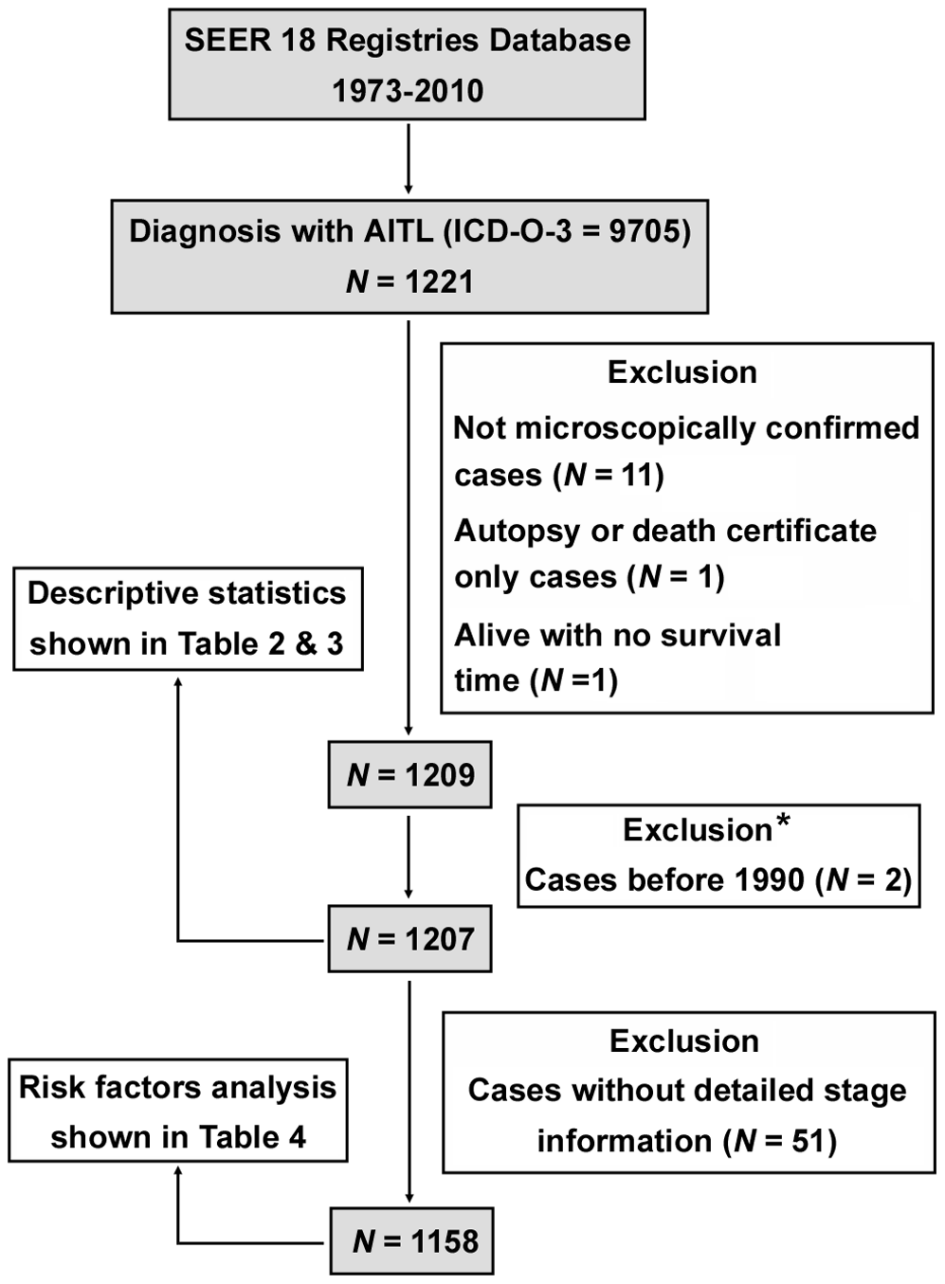
Patient selection flowchart. ICD-O-3, International Classification of Diseases for Oncology, 3rd edition; AITL, angioimmunoblastic T-cell lymphoma; SEER, Surveillance, Epidemiology, and End Results. *The histology code 9705 first appeared as a code for AITL in ICD-O-2, which was published in 1990 [20]. We excluded 2 cases diagnosed before 1990, which might be found late and abstracted after the introduction of the ICD-O-2 codes.

This retrospective study was conducted using the SEER public-use database 1973 to 2010 (November 2012 Submission) [19]. The SEER Program, sponsored by the National Cancer Institute, currently collects and publishes cancer incidence and survival data from 18 population-based cancer registries covering approximately 27.8% of the United States population. For more information about SEER, one is referred to its Web site (http://seer.cancer.gov/). Patients with AITL were identified using the third edition of the International Classification of Diseases for Oncology (ICD-O-3) histology code 9705. We obtained the following patient-specific data: age at diagnosis, sex, race, stage, year of diagnosis, diagnostic confirmation, vital status recode, length of survival, and cause of death. We excluded cases without microscopically confirmed diagnosis, those identified only through autopsy or death certificate, and those without follow-up records. For analyses requiring stratification by stage of disease, patients for whom no stage information was provided were excluded. Figure 1 outlines the inclusion process that was used to select cases with AITL for analysis.

The study was performed in accordance with the Declaration of Helsinki. Approval was obtained from the Institutional Review Board of the First Affiliated Hospital of Nanjing Medical University.

### Outcome assessment

In current study, the follow-up cutoff was December 31, 2010. OS was calculated from the date of diagnosis to the date of death from any cause or the follow-up cutoff. Disease-specific survival (DSS) was calculated from the date of diagnosis to the date of death from non-Hodgkin's lymphoma or the follow-up cutoff. Patients who died from causes other than non-Hodgkin's lymphoma were considered censored at their date of death in DSS analysis.

### Statistical Analysis

For survival analysis, the whole period of evaluation was segmented into five consecutive time periods based on date of diagnosis (1992 to 1998, 1999 to 2001, 2002 to 2004, 2005 to 2007, and 2008 to 2010). For the November 2012 submission, the survival time was calculated in months using complete dates (month, day, and year components) and the results were always rounded down. One hundred and twenty cases had a SEER-assigned survival time of 0 months after the exclusion process described above. To minimize any bias that could result from excluding these cases from the analysis, we assigned each case with zero survival time a survival time of 0.5 month. Survival outcomes were estimated using the Kaplan-Meier method. Log-rank test was used to compare survival distributions. We also studied the prognostic effect of the various clinical variables using univariate and multivariate Cox proportional hazards models. All statistical analysis was performed using STATA/SE 12.0 (Stata Corp, College Station, TX). Results were considered significant if a two-sided *P*<0.05 was obtained.

## Results

### Patient characteristics

From 1973 through 2010, 1221 AITL cases were ascertained by the SEER program. A total of 1207 patients with AITL matching the specified criteria described in Figure 1 were included in the final sample for this investigation. Demographic and clinical data for the entire study population are presented in Table 2.

**Table 2:** Patient Characteristics (N = 1207).

Characteristic	No. of Patients	%
**Age, years**		
Median (range)	69 (10 to 96)	
10 or less	1	0.1
11–20	1	0.1
21–30	12	1.0
31–40	36	3.0
41–50	110	9.1
51–60	238	19.7
61–70	262	21.7
71–80	357	29.6
81–90	179	14.8
91 or more	11	0.9
**Sex**		
Male	622	51.5
Female	585	48.5
**Race**		
White	980	81.2
Black	90	7.5
Asian/Pacific Islander	131	10.9
Other or unknown	6	0.5
**Stage**		
I	101	8.4
II	98	8.1
III	507	42.0
IV	452	37.5
Unstaged	49	4.1
**Year of diagnosis**		
1992–1998	101	8.4
1999–2001	127	10.5
2002–2004	241	20.0
2005–2007	327	27.1
2008–2010	411	34.1

AITL is generally a disease of the elderly, with a median age at diagnosis of 69 years (range, 10 to 96 years). Of the cohort of patients, 51.5% were male, and 48.5% female. The racial/ethnic distribution of this study was 81.2% white, 10.9% Asian/Pacific Islander, 7.5% black, and 0.5% other or unknown. At presentation, the overwhelming majority of patients (79.5%) had an advanced-stage (III to IV) disease by Ann Arbor classification. The proportion of patients diagnosed in 1992 to 1998, 1999 to 2001, 2002 to 2004, 2005 to 2007, and 2008 to 2010 was 8.4%, 10.5%, 20.0%, 27.1%, and 34.1% respectively.

### Survival Outcomes

The median follow-up of the study was 13 months (range, 0 to 220 months). Among 1207 AITL cases, 742 deaths were observed as of December 31, 2010. Of these, 552 (74.4%) were because of non-Hodgkin's lymphoma and were considered disease-specific deaths. The second major cause of death (5.1%) was heart disease.

**Figure 2:**
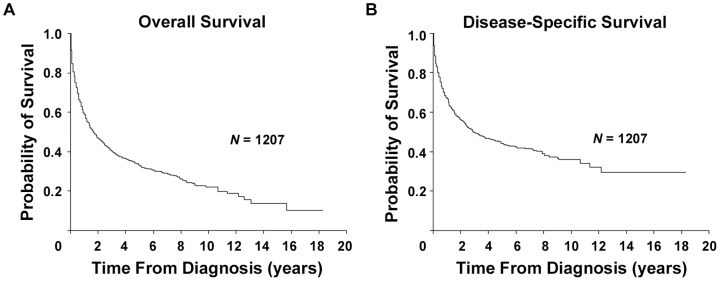
Survival of patients with angioimmunoblastic T-cell lymphoma. OS (A) and DSS (B).

Overall survival probabilities at 2, 5 and 10 years were 46.8% (95% CI, 43.8% to 49.8%), 32.9% (95% CI, 29.8% to 36.0%), and 21.9% (95% CI, 18.2% to 25.9%), respectively. Two-year, 5-year, and 10-year DSS rates were 56.1% (95% CI, 52.9% to 59.1%), 44.0% (95% CI, 40.5% to 47.5%), and 35.9% (95% CI, 31.3% to 40.5%), respectively (Figure 2). Table 3 reflects the OS and DSS according to the demographic and clinical characteristics of AITL patients.

**Table 3:** Overall and Disease-Specific Survival (N = 1207).

Characteristic	Overall Survival			Disease-Specific Survival		
	2 Year, % (95% CI)	5 Year, % (95% CI)	10 Year, % (95% CI)	2 Year, % (95% CI)	5 Year, % (95% CI)	10 Year, % (95% CI)
**All**	46.8 (43.8 to 49.8)	32.9 (29.8 to 36.0)	21.9 (18.2 to 25.9)	56.1 (52.9 to 59.1)	44.0 (40.5 to 47.5)	35.9 (31.3 to 40.5)
**Age, years**						
50 or less	67.0 (58.9 to 73.9)	57.5 (48.7 to 65.3)	47.6 (36.7 to 57.7)	74.4 (66.3 to 80.9)	64.6 (55.4 to 72.4)	58.6 (47.8 to 68.0)
51–60	54.5 (47.5 to 60.9)	38.8 (31.3 to 46.2)	31.0 (22.2 to 40.3)	62.7 (55.5 to 69.0)	48.4 (40.1 to 56.2)	40.7 (30.0 to 51.2)
61–70	52.7 (45.9 to 59.1)	39.6 (32.5 to 46.6)	19.5 (11.2 to 29.5)	57.4 (50.5 to 63.8)	46.7 (39.2 to 53.9)	31.5 (21.3 to 42.3)
71–80	39.7 (34.4 to 45.0)	22.9 (18.0 to 28.2)	12.5 (7.6 to 18.7)	49.8 (43.9 to 55.3)	35.9 (29.6 to 42.3)	30.3 (22.4 to 38.6)
81 or more	25.1 (18.8 to 31.7)	14.4 (9.0 to 21.0)	12.3 (6.9 to 19.5)	40.6 (32.2 to 48.8)	29.9 (21.3 to 39.0)	25.6 (15.6 to 36.9)
**Sex**						
Male	44.1 (40.0 to 48.2)	28.1 (24.0 to 32.3)	19.3 (14.8 to 24.3)	54.2 (49.8 to 58.5)	40.7 (35.8 to 45.5)	32.0 (25.8 to 38.3)
Female	49.7 (45.4 to 53.9)	38.3 (33.7 to 42.9)	24.9 (18.9 to 31.4)	58.0 (53.5 to 62.3)	47.6 (42.5 to 52.5)	40.6 (34.0 to 47.1)
**Race**						
White	47.6 (44.2 to 50.8)	33.2 (29.7 to 36.7)	22.4 (18.2 to 26.9)	56.9 (53.4 to 60.2)	44.0 (40.1 to 47.9)	35.8 (30.6 to 41.0)
Other	43.7 (36.9 to 50.3)	31.4 (24.6 to 38.4)	19.8 (12.4 to 28.6)	52.7 (45.4 to 59.6)	43.9 (35.9 to 51.6)	36.0 (25.8 to 46.3)
**Stage**						
I	50.5 (40.1 to 60.0)	43.7 (33.3 to 53.6)	34.7 (22.6 to 47.0)	66.0 (54.8 to 75.0)	62.4 (50.7 to 72.0)	56.1 (39.8 to 69.6)
II	63.3 (52.3 to 72.4)	39.5 (27.6 to 51.2)	24.7 (10.7 to 41.7)	76.5 (65.5 to 84.4)	56.8 (43.1 to 68.4)	47.3 (26.8 to 65.4)
III	45.5 (40.9 to 50.0)	32.5 (27.8 to 37.4)	23.0 (17.6 to 28.8)	54.4 (49.5 to 59.1)	44.1 (38.7 to 49.4)	35.1 (28.0 to 42.2)
IV	42.2 (37.3 to 47.1)	28.3 (23.5 to 33.3)	18.7 (13.2 to 25.0)	49.1 (43.8 to 54.1)	36.3 (30.7 to 41.8)	30.2 (23.3 to 37.4)
Unstaged	60.1 (44.8 to 72.5)	40.1 (25.0 to 54.8)	16.7 (4.1 to 36.7)	74.8 (58.9 to 85.3)	52.5 (33.9 to 68.2)	39.4 (19.6 to 58.7)
**Year of diagnosis**						
1992–1998	44.6 (34.7 to 53.9)	28.7 (20.3 to 37.7)	15.8 (9.5 to 23.6)	53.1 (42.5 to 62.7)	41.7 (31.2 to 51.8)	25.4 (16.2 to 35.6)
1999–2001	51.2 (42.2 to 59.5)	35.4 (27.2 to 43.7)	26.0 (18.7 to 33.8)	60.4 (50.8 to 68.7)	46.7 (37.2 to 55.8)	43.3 (33.7 to 52.5)
2002–2004	42.7 (36.4 to 48.9)	27.8 (22.3 to 33.6)	NR	55.2 (48.3 to 61.5)	41.6 (34.7 to 48.4)	NR
2005–2007	48.9 (43.4 to 54.2)	37.7 (31.9 to 43.6)	NR	59.1 (53.2 to 64.5)	48.5 (41.9 to 54.8)	NR
2008–2010	45.1 (38.8 to 51.2)	NR	NR	51.2 (44.6 to 57.4)	NR	NR

**Abbreviations**: NR, not reached.

### Prognostic factors

Analyses of prognostic factors are listed in Table 4. On univariate analysis, older age and advanced-stage (III to IV) disease were associated with an increased risk of all-cause mortality and disease-specific death.

**Table 4:** Cox Proportional Hazards Regression Analysis for Overall and Disease-Specific Survival (N = 1158).

Variable	Univariate Analysis						Multivariate Analysis					
	Overall Survival			Disease-Specific Survival			Overall Survival			Disease-Specific Survival		
	HR	95% CI	*P*	HR	95% CI	*P*	HR	95% CI	*P*	HR	95% CI	*P*
**Age, years**												
50 or less	0.7	0.5 to 0.9	0.012	0.7	0.5 to 0.9	0.018	0.7	0.5 to 0.9	0.011	0.7	0.5 to 0.9	0.017
51–60	Reference			Reference			Reference			Reference		
61–70	1.1	0.9 to 1.4	0.442	1.2	0.9 to 1.5	0.267	1.1	0.9 to 1.4	0.460	1.2	0.9 to 1.5	0.304
71–80	1.7	1.4 to 2.1	<0.001	1.6	1.2 to 2.0	<0.001	1.7	1.4 to 2.1	<0.001	1.6	1.2 to 2.0	0.001
81 or more	2.5	2.0 to 3.2	<0.001	2.2	1.6 to 2.9	<0.001	2.7	2.1 to 3.4	<0.001	2.3	1.7 to 3.0	<0.001
**Sex**												
Male	1.1	1.0 to 1.3	0.107	1.1	0.9 to 1.3	0.348	1.3	1.1 to 1.5	0.003	1.2	1.0 to 1.4	0.041
Female	Reference			Reference			Reference			Reference		
**Race**												
White	0.9	0.8 to 1.1	0.328	0.9	0.7 to 1.1	0.421	0.8	0.7 to 1.0	0.064	0.8	0.7 to 1.0	0.113
Other	Reference			Reference			Reference			Reference		
**Stage**												
I to II	Reference			Reference			Reference			Reference		
III to IV	1.4	1.1 to 1.7	0.001	1.8	1.4 to 2.4	<0.001	1.5	1.2 to 1.8	<0.001	1.9	1.5 to 2.5	<0.001
**Year of diagnosis**												
1979–1998	1.3	1.0 to 1.7	0.097	1.4	1.0 to 2.0	0.064	1.3	1.0 to 1.8	0.058	1.5	1.0 to 2.1	0.033
1999–2001	Reference			Reference			Reference			Reference		
2002–2004	1.2	0.9 to 1.6	0.135	1.2	0.9 to 1.6	0.276	1.3	1.0 to 1.6	0.073	1.2	0.9 to 1.7	0.188
2005–2007	1.0	0.8 to 1.3	0.832	1.0	0.7 to 1.4	0.997	1.0	0.8 to 1.3	0.952	1.0	0.8 to 1.4	0.808
2008–2010	1.1	0.8 to 1.4	0.695	1.2	0.9 to 1.6	0.284	1.1	0.8 to 1.4	0.647	1.2	0.9 to 1.6	0.291

**Abbreviations**: HR, hazard ratio.

On multivariate analysis, no statistically significant differences in OS (*P* = 0.460) and DSS (*P* = 0.304) were identified between patients diagnosed at 51 to 60 years and those at 61 to 70 years. Cases age 50 years or younger had a significantly lower incidence of all-cause (hazard ratio [HR], 0.7; 95% CI, 0.5 to 0.9; *P* = 0.011) and disease-specific mortality (HR, 0.7; 95% CI, 0.5 to 0.9; *P* = 0.017) than did those diagnosed between the ages of 51 and 60 years. Patients diagnosed at 71 to 80 years had a markedly higher risk of all-cause (HR, 1.7; 95% CI, 1.4 to 2.1; *P*<0.001) and disease-specific death (HR, 1.6; 95% CI, 1.2 to 2.0; *P* = 0.001) than did those in the 51- to 60-year age category.

Although having no influence on survival in univariate analysis, male sex conferred a significantly worse OS (HR, 1.3; 95% CI, 1.1 to 1.5; *P* = 0.003) and DSS (HR, 1.2; 95% CI, 1.0 to 1.4; *P* = 0.041) in multivariate analysis. Patients with advanced-stage disease had a markedly higher risk of all-cause (HR, 1.5; 95% CI, 1.2 to 1.8; *P*<0.001) and disease-specific death (HR, 1.9; 95% CI, 1.5 to 2.5; *P*<0.001) in comparison to those with early stage disease.

Of note, no significant differences in OS were observed with more modern treatment eras in our multivariable model (all *P*>0.05). It seems that patients diagnosed during the 1992–1998 period experienced a reduced DSS (HR, 1.5; 95% CI, 1.0 to 2.1; *P* = 0.033) compared with the baseline group (1999–2001 period). However, when performing joint test to determine the overall effect of the 5 categorical variables of treatment eras, we cannot reject the null hypothesis that all of the coefficients are zero (joint significance test, *P* = 0.138), indicating the absence of changes in AITL patient survival over time.

## Discussion

Although recognized as one of the most common forms of PTCL, AITL is a rare clinicopathologic entity according to the 2008 WHO classifications, with an estimated incidence of 0.05 cases per 100 000 person-years in the United States [21]. So far, limited data are available on clinical features, survival patterns, and prognostic factors in AITL. To the best of our knowledge, the current study represents the largest specific series of patients with AITL and the first investigation on temporal changes in survival of AITL patients.

Numerous clinical and pathologic classification schemes have been introduced over the past 3 decades, particularly for non-Hodgkin lymphomas. The histology code 9705 first appeared as a code for AITL in the second edition of the International Classification of Diseases for Oncology (ICD-O-2), which was published in 1990 [20]. In the current study, we excluded 2 cases diagnosed before 1990, which might be found late and abstracted after the introduction of the ICD-O-2 codes.

The clinicobiologic features of AITL observed in our study were similar to those described by other researchers [16]–[18]. AITL chiefly affected elderly adults in their seventh (21.7%) or eighth (29.6%) decades, at a median age of 69 years. The incidence in males (51.5%) was slightly higher than that in females (48.5%). Eighty percent of patients presented with advanced-stage (III to IV) disease. Of 1207 patients, most cases were diagnosed during or after the year 2002 (81%).

AITL is an aggressive neoplasm with a poor survival outcome. In 157 AITL patients retrieved from the Groupe d'Etude des Lymphomes de l'Adulte (GELA) trials [16], the 2-, 5-, and 7-year OS rates were 51%, 33%, and 29% respectively. Tokunaga et al described a multicenter retrospective study of 207 AITL patients in Japan [17]. In their analysis, the 3-, 5-, and 7-year OS rates were 54%, 41%, and 35% respectively. More recently, Federico et al presented the data of 243 AITL cases from the International Peripheral T-Cell Lymphoma Project, reporting an OS of 33% at 5 years [18].

In the current study, we showed that the OS rates at 2, 5 and 10 years were 46.8%, 32.9%, and 21.9% respectively, which were similar to those reported by previous researches mentioned above. Interestingly, our 5-year OS probability was almost the same as those in GELA study and International Peripheral T-Cell Lymphoma Project, with a narrower 95% CI (25.6% to 41.0% in GELA trials vs 29.8% to 36.0% in the present study). Our results, together with those from other researchers, confirmed the dismal prognosis of AITL with current treatment strategies.

A number of clinicobiologic and pathological characteristics, including age, stage, systemic symptoms, skin rush/pruritus, edema, ascites, lactate dehydrogenase, hemoglobin [14], lymphocyte value [22], presence of clear and convoluted cells [23], achievement of complete remission [24], [25], drug exposure history [25] and cytogenetic findings [26], have been reported to be associated with survival of AITL patients, with controversial results. The International Prognostic Index (IPI), the widely used predictive model for many types of non-Hodgkin lymphoma, was shown to be significantly related to treatment outcomes of AITL patients [17]. A recently proposed prognostication score system for PTCL, not otherwise specified (PIT) also seemed to impact survival of patients with AITL [17]. However, the prognosis significance of both models for AITL have not been be supported by other investigations [3], [16], [18], [27], [28]. In report from GELA trials, only male sex, mediastinal lymphadenopathy, and anemia were suggestive of decreased survival [16]. Tokunaga et al showed that age older than 60 years, elevated white blood cell and IgA levels, the presence of anemia and thrombocytopenia, and extranodal involvement at more than 1 site adversely affected OS [17]. Federico et al recently suggested a new predictive model for AITL (PIAI), using five significant prognostic factors: age>60 years, PS>2, extranodal sites more than one, B symptoms, and platelet count<150×10^9^/L. They showed that the novel prognostic index system did better compared with IPI and PIT in identifying AITL patient subsets with different outcomes [18].

In the present study, older age, advanced-stage disease and male sex were identified adverse predictors for OS and DSS of AITL patients. Patients diagnosed at 71 to 80 years had a 70% higher risk of death than did those at 51 to 60 years, while cases age 50 years or younger had a 30% lower risk of mortality compared with the reference group. However, we failed to find any difference in survival outcome between patients diagnosed at 51 to 60 years and those at 61 to 70 years. Advanced-stage (III to IV) disease was associated with a 50% higher risk of death compared with early stage lymphoma. In addition, we demonstrated in the largest cohorts of cases to date that male sex is an independent poor prognostic marker for patients with AITL (*P* = 0.003 and *P* = 0.041 for OS and DSS, respectively), which is consistent with the results from GELA study.

In the present study, older age, advanced-stage disease and male sex were identified adverse predictors for OS and DSS of AITL patients. Patients diagnosed at 71 to 80 years had a 70% higher risk of death than did those at 51 to 60 years, while cases age 50 years or younger had a 30% lower risk of mortality compared with the reference group. However, we failed to find any difference in survival outcome between patients diagnosed at 51 to 60 years and those at 61 to 70 years. Advanced-stage (III to IV) disease was associated with a 50% higher risk of death compared with early stage lymphoma. In addition, we demonstrated in the largest cohorts of cases to date that male sex is an independent poor prognostic marker for patients with AITL (*P* = 0.003 and *P* = 0.041 for OS and DSS, respectively), which is consistent with the results from GELA study.

An important finding in our study is the trend toward better OS for white patients in comparison to other ethnic groups (*P* = 0.064). To our knowledge, our report represents the first study addressing the prognostic role of race/ethnicity in AITL patients, while comprehensively controlling for other variables. Actually, there has been a large body of data describing racial and economic disparities in cancer treatment and outcomes over the last decade. Poor prognosis for black compared with white patients was reported across many types of malignancy. Underuse of effective treatment has been indicated to play a key role for this difference in survival among ethnic groups [29].

Various strategies have been proposed for the management of AITL, ranging from the watch-and-wait attitude to intensive chemotherapy followed by ASCT. However to date, no convincing results from large series of randomized trials have demonstrated that one treatment is superior to others. Our large sample size allowed us to examine the temporal trends in survival of patients with AITL, with acceptable statistical power. In the present study, we failed to find any survival rate differences among subgroups diagnosed in the 5 periods spanning 19 years, indicating the lack of survival improvement after the sequential attempts made for AITL treatment. Thus, novel management approaches are warranted.

Several groups have reported the benefit of high dose chemotherapy with ASCT (HDT-ASCT) for AITL patients. Data from the Lymphoma Working Party of the European Group for Blood and Marrow Transplantation (EBMT), which included 146 AITL patients treated with HDT-ASCT, reported a 59% OS and 42% progression-free survival at 4 years after transplantation. Best survival outcomes were observed in those patients who underwent ASCT during first complete remission [10]. Limited data so far are available regarding the use of alloSCT in AITL patients. A recently published retrospective study from the Lymphoma Working Party of EBMT showed encouraging results. Forty-five patients with AITL underwent an alloSCT, with a median age of 48 years. Of those, 25 patients underwent a myeloablative alloSCT, while 20 underwent a reduced-intensity alloSCT. OS and PFS rates at 3 years were 64% and 53%, respectively. Relapse rate was estimated as 16% and 20% at 2 and 3 years, respectively [13]. Prospective multicenter clinical trials are much needed to determine the role of ASCT and alloSCT in AITL.

Clinical outcomes among AITL patients remain dismal in part because of the lack of understanding of genetics of this disease. Odejide et al recently showed that AITL is characterized by high frequencies of overlapping mutations in epigenetic modifiers, including TET2, IDH2 and DNMT3A. This study defines the first genetic landscape of AITL across over 200 genes known to be recurrently mutated in hematologic neoplasms. The complement of genes frequently mutated in AITL resembles myeloid diseases more than other lymphomas, which could help explain the poor outcomes after treatment with regimens developed against B cell lymphomas [30].

A major concern for the epidemiological cancer study based on information from population-based cancer registries is diagnosis reliability. SEER collects information on incidence, prevalence, and survival from specific geographic areas representing 27.8% of the US population. Thus, cancers may have been diagnosed and classified by numerous independent pathologists, perhaps using diverse methodologies. Excellent agreement in diagnosis between the expert review and the SEER registry record has been reported for small-cell carcinoma of the lung. Agreement of registry and expert diagnoses was nearly 80% for the most common non-Hodgkin's lymphoma (NHL) subtype, diffuse large B cell lymphoma. Another subtype of NHL, chronic lymphocytic leukemia, might be underreported in certain cancer registries [31], [32].

The SEER program registries routinely collect data on patient demographics, primary tumor site, tumor morphology and stage at diagnosis, first course of treatment, and follow-up for vital status. Unfortunately, detailed treatment data are not collected by the SEER. Since there is a known heterogeneity in the management of AITL, this limitation made it impossible to evaluate interactions between clinical outcome and specific treatment modalities used for AITL directly. Moreover, the SEER database does not provide enough clinicopathologic information needed for calculating IPI and PIT scores. Therefore, we failed to analyze survival outcomes according to these prognostic models, although several previous studies showed that both index systems were not predictive of survival for AITL patients [3], [16], [18], [27], [28]. Nevertheless, the present study provides novel information on AITL and the grounds for further research.

In conclusion, AITL is a rare lymphoid neoplasm with dismal prognosis, even when treated intensively. There has been no survival improvement for patients with AITL over the past two decades. Further investigations are warranted to develop more effective treatment for this disease.
